# Supplementary material for: Can vaccination roll-out be more equitable if population risk is taken into account?
Source: PLoS One. 2021 Nov 15;16(11):e0259990. doi: 10.1371/journal.pone.0259990 (PMC8592495; doi:10.1371/journal.pone.0259990)
Supplement: S1 File — (PDF) [file pone.0259990.s001.pdf]

S1 File: Variables included in the frailty index. Variables from the English Longitudinal Survey of Ageing (ELSA).

**No**	**Variable**
Mobility	
1	Difficulty walking 100 yards
2	Difficulty sitting for 2 hours
3	Difficulty getting up from chair after sitting long periods
4	Difficulty climbing several flights stairs without resting
5	Difficulty climbing one flight stairs without resting
6	Difficulty stooping, kneeling, or crouching
7	Difficulty reaching or extending arms above shoulder level
8	Difficulty pulling or pushing large objects
9	Difficulty lifting or carrying weights over 10 pounds
10	Difficulty picking up 5p coin from the table
Activities Daily Living (ADL)	
11	Difficulty dressing, including putting on shoes and socks
12	Difficulty walking across the room
13	Difficulty bathing or showering
14	Difficulty eating, such as cutting up food
15	Difficulty getting in and out of bed
16	Difficulty using the toilet, including getting up or down
Instrumental Activities Daily Living (IADL)	
17	Difficulty using map to figure out how to get around strange
18	Difficulty preparing a hot meal
19	Difficulty shopping for groceries
20	Difficulty making telephone calls
21	Difficulty taking medications
22	Difficulty managing money, such as paying bills and keeping tracks of expenses
23	Difficulty doing work around the house or garden

Self-reported general health	
24	Self-reported general health
Ever diagnosed by health professionals	
25	Hypertension
26	Angina
27	Heart attack
28	Congestive heart failure
29	Abnormal heart rhythm
30	Diabetes/high blood sugar
31	Stroke
32	Lung diseases
33	Asthma
34	Arthritis
35	Osteoporosis
36	Cancer
37	Parkinson diseases
38	Psychiatric conditions
39	Alzheimer diseases
40	Dementia
Subjective sensory function	
41	Poor or fair self-reported eyesight
42	Poor or fair self-reported hearing
Falls and other health issues	
43	Fallen down
44	Fractured hip
45	Had joint replacement
Cognitive function	
46	Cannot answer correct day of month
47	Cannot answer correct month
48	Cannot answer correct year

49	Cannot answer correct day
50	Immediate recall
51	Delayed recall

All binary variables are recoded, using the convention that ‘0’ indicates absence and ‘1’ presence of a deficit. For each person, deficit points are summed and divided by the total number of deficits, to produce a frailty index score with a range from 0 to 1.
