# Supplementary material for: Can vaccination roll-out be more equitable if population risk is taken into account?
Source: PLoS One. 2021 Nov 15;16(11):e0259990. doi: 10.1371/journal.pone.0259990 (PMC8592495; doi:10.1371/journal.pone.0259990)
Supplement: S2 File — (PDF) [file pone.0259990.s002.pdf]

S2 File: Statistical analysis

Missing data in survey results for the frailty index data (S1 File) was imputed by Multiple Imputations by Chained Equations, using Stata's *Multiple Imputation* library [1]. Twenty imputations were used.

A generalised ordinal logistic regression on each survey respondent's frailty against their sex, age group, and area deprivation quintile was used to estimate the probability of pre-frailty and frailty for a given sex, age range and area deprivation quintile. Generalised ordinal logistic regression was performed using Stata's *gologit2* programme [2, 3].

Age groups were categorised as: 50-54, 55-59, 60-64, 65-69, 70-74, 75-79, 80-84, 85-89, ≥90.

Sex was categorised as male or female.

Area deprivation quintile was categorised using English Indices of Multiple Deprivation. (IMD), with quintile 1 the least advantaged areas (i.e. most deprived) and quintile 5 the most advantages areas (i.e. least deprived).

Table A2: Results of generalised ordinal logistic regression			
	**Coefficient**	**95% confidence. interval - lower**	**95% confidence interval - upper**
**Pre-frail**			
Index Multiple Deprivation, reference: 5th quintile (most advantaged areas)			
1st quintile (least advantaged areas)	1.43	1.17	1.69
2nd	0.63	0.39	0.86
3rd	0.42	0.20	0.65
4th	0.15	-0.07	0.38
Age *groups, reference: 50-54 years old*			
55-59	0.02	-0.57	0.62
60-64	0.74	0.23	1.25
65-69	0.80	0.29	1.30
70-74	1.25	0.76	1.75
75-79	1.57	1.07	2.07
80-84	2.02	1.52	2.53
85-89	2.80	2.26	3.33
≥ 90	3.48	2.87	4.10

Sex, reference: males			
Female	0.37	0.22	0.52
Constant	-3.32	-3.83	-2.81
**Frail**			
Index Multiple Deprivation, reference: 5th quintile (most advantaged areas)			
1st quintile (least advantaged areas)	1.46	1.13	1.79
2nd	0.84	0.49	1.19
3rd	0.54	0.22	0.87
4th	0.09	-0.25	0.42
Age *groups, reference: 50-54 years old*			
55-59	0.46	-0.42	1.34
60-64	0.97	0.19	1.74
65-69	0.82	0.06	1.59
70-74	1.13	0.37	1.89
75-79	1.39	0.63	2.16
80-84	1.78	1.02	2.54
85-89	2.42	1.64	3.19
≥ 90	3.32	2.50	4.13
Sex, reference: males			
Females	0.33	0.12	0.55
Constant	-4.37	-5.18	-3.57

References

1. StataCorp, *Stata Multiple-Imputation Reference Manual*. 16 ed. Vol. 1. 2019, College Station, TX: Stata Press.
2. Williams, R., *Generalized ordered logit/partial proportional odds models for ordinal dependent variables.* Stata Journal, 2006. **6**(1): p. 58-82.
3. Williams, R., *GOLOGIT2: Stata module to estimate generalized logistic regression models for ordinal dependent variables.* 2019.
