# Supplementary material for: Can vaccination roll-out be more equitable if population risk is taken into account?
Source: PLoS One. 2021 Nov 15;16(11):e0259990. doi: 10.1371/journal.pone.0259990 (PMC8592495; doi:10.1371/journal.pone.0259990)

S3 File: Number of first vaccine doses administered, relative to the number of people diagnosed with a range of risk factors, in each Clinical Commissioning Group area.

The number of people diagnosed with each condition in each Clinical Commissioning Group are sourced from Public Health England's Public Health Profiles [https://fingertips.phe.org.uk/]

Fig S3a Number of first vaccine doses administered in each Clinical Commissioning Group area relative to the number of people diagnosed with atrial fibrillation in that area (on 25 February 2021).

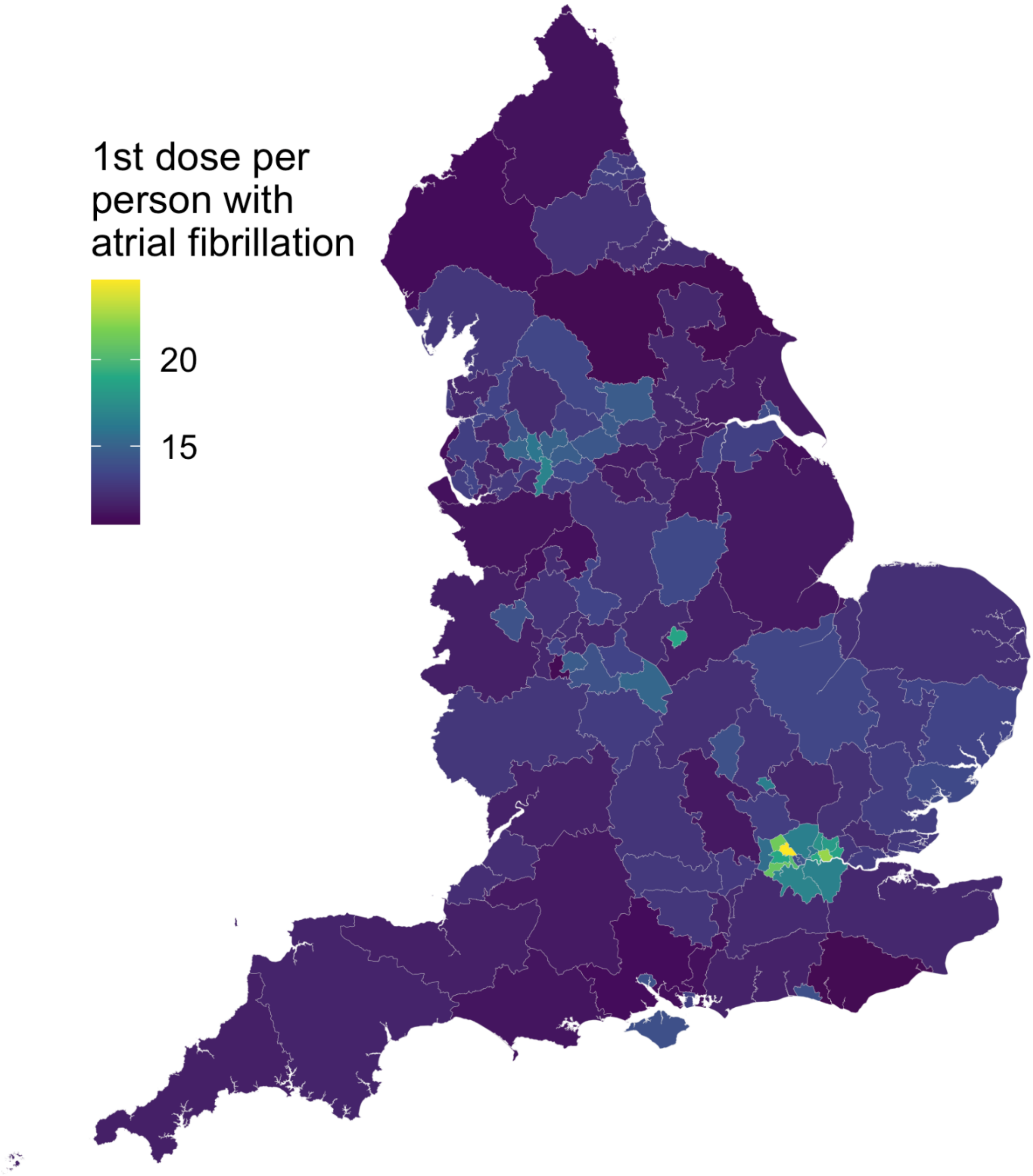

Fig S3b Number of first vaccine doses administered in each Clinical Commissioning Group area relative to the number of people diagnosed with chronic kidney disease in that area (on 25 February 2021).

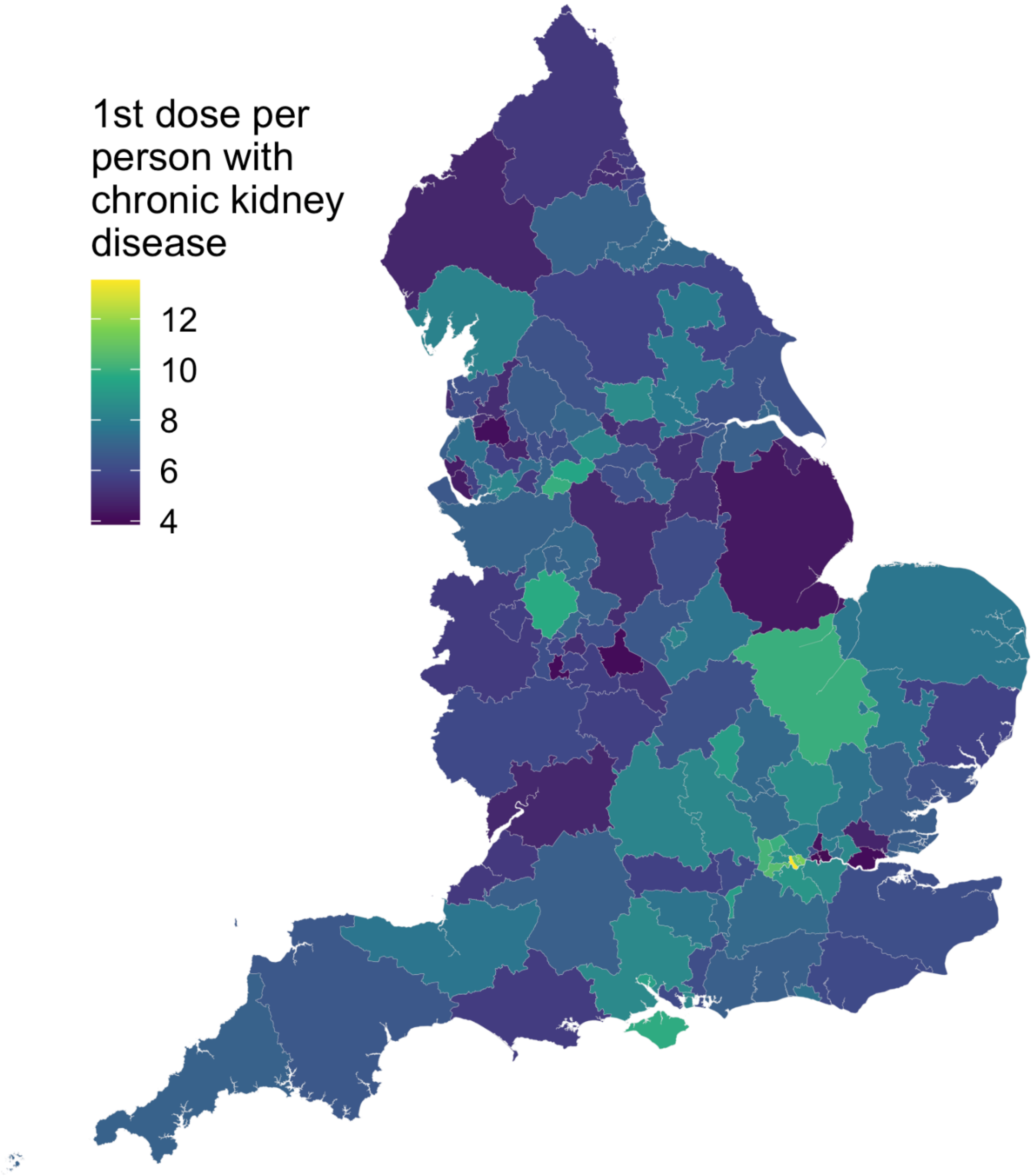

Fig S3c Number of first vaccine doses administered in each Clinical Commissioning Group area relative to the number of people diagnosed with diabetes in that area (on 25 February 2021).

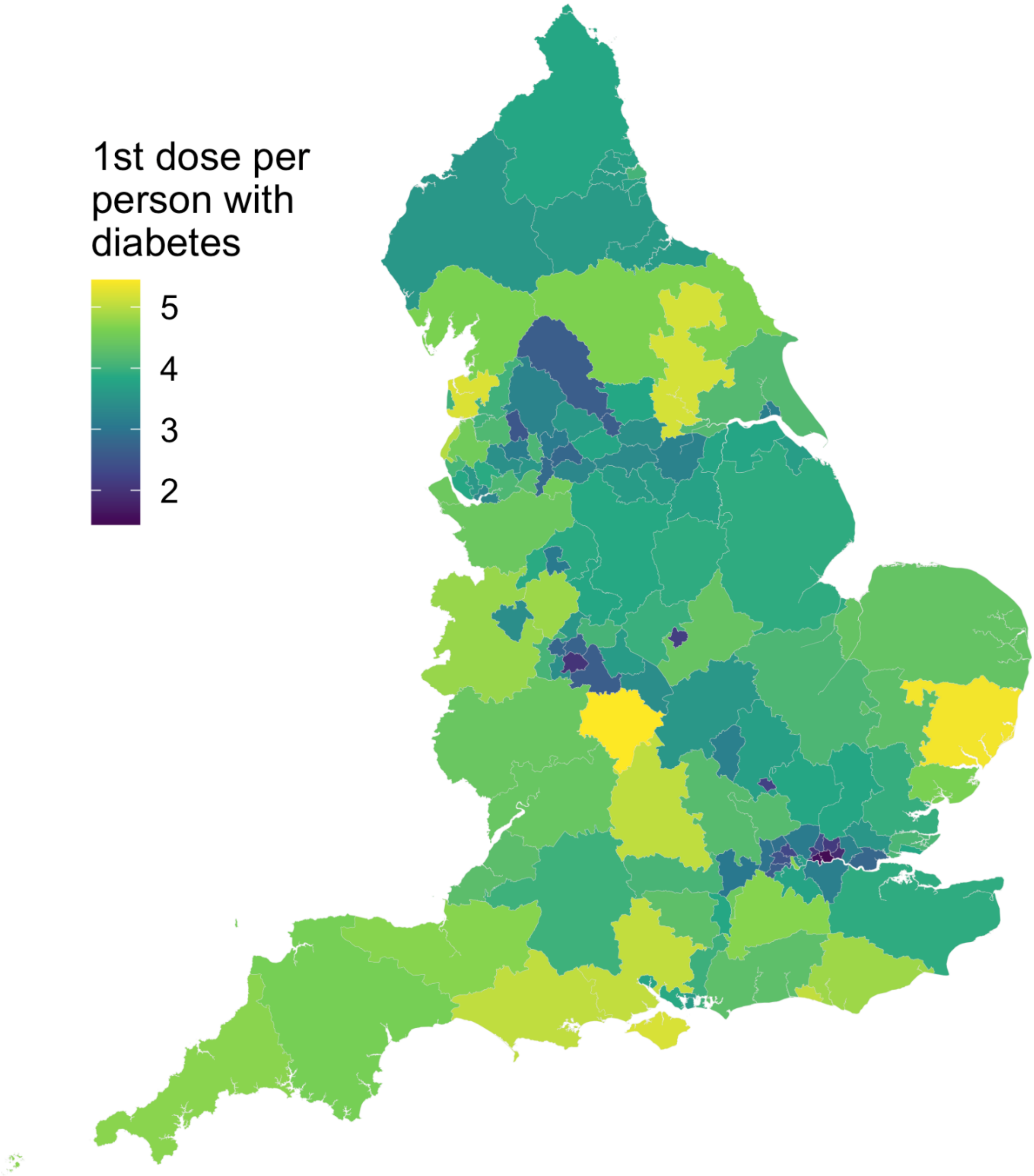

Fig S3d Number of first vaccine doses administered in each Clinical Commissioning Group area relative to the number of people diagnosed with a learning disability in that area (on 25 February 2021).

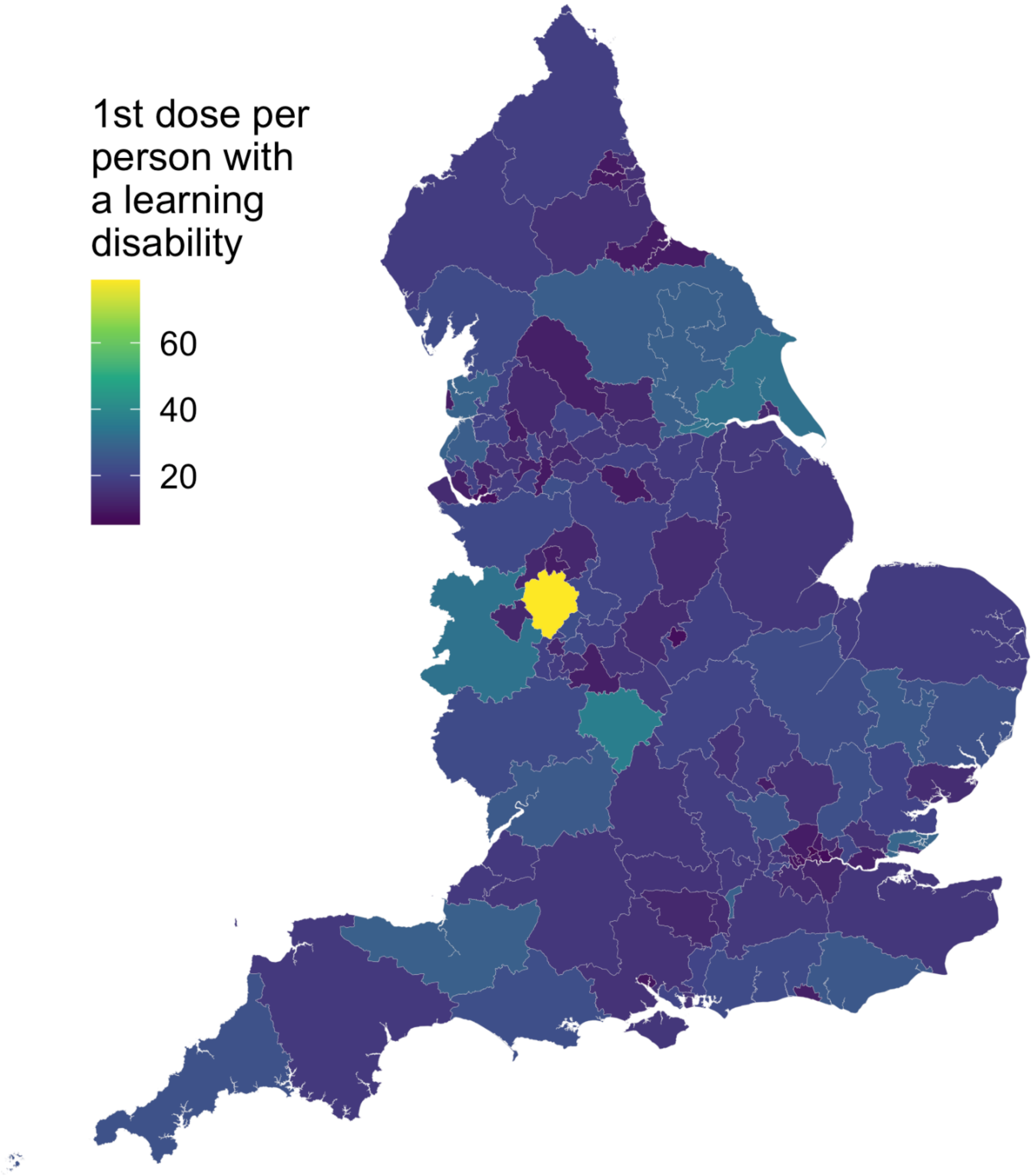

Fig S3e Number of first vaccine doses administered in each Clinical Commissioning Group area relative to the number of people with obesity in that area (on 25 February 2021).

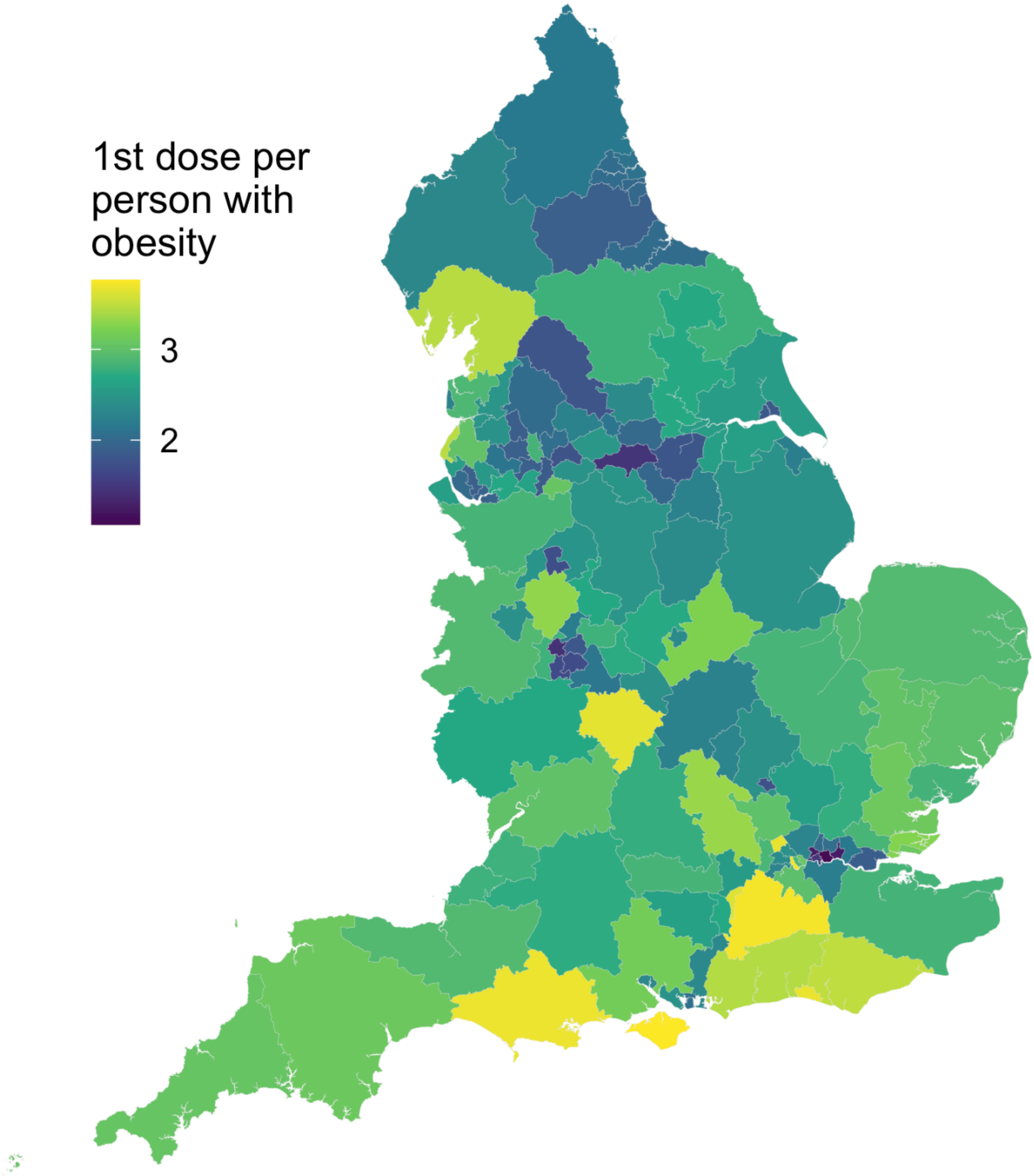

Fig S3f Number of first vaccine doses administered in each Clinical Commissioning Group area relative to the number of smokers in that area (on 25 February 2021).

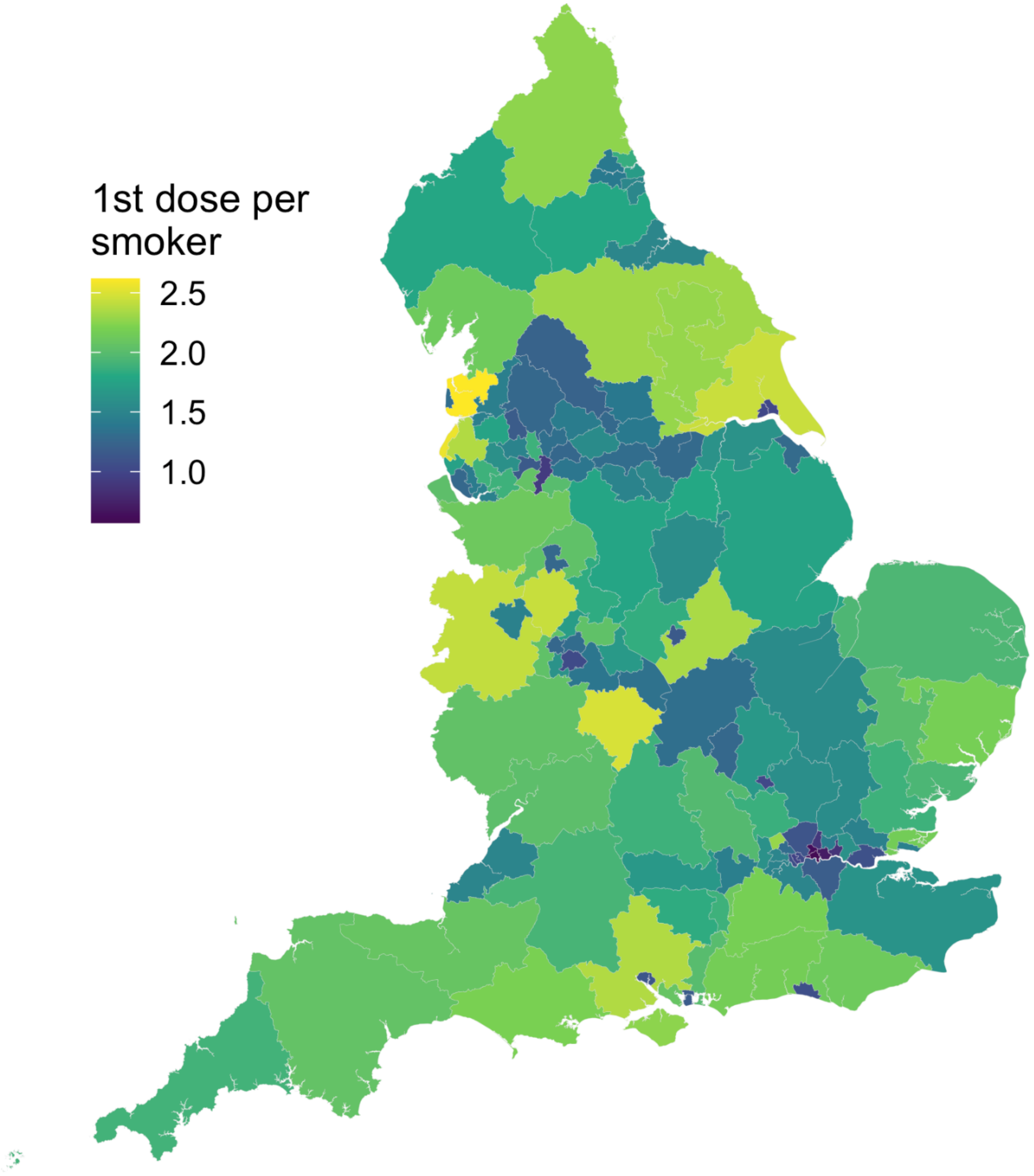

Fig S3g Number of first vaccine doses administered in each Clinical Commissioning Group area relative to the number of former smokers in that area (on 25 February 2021).

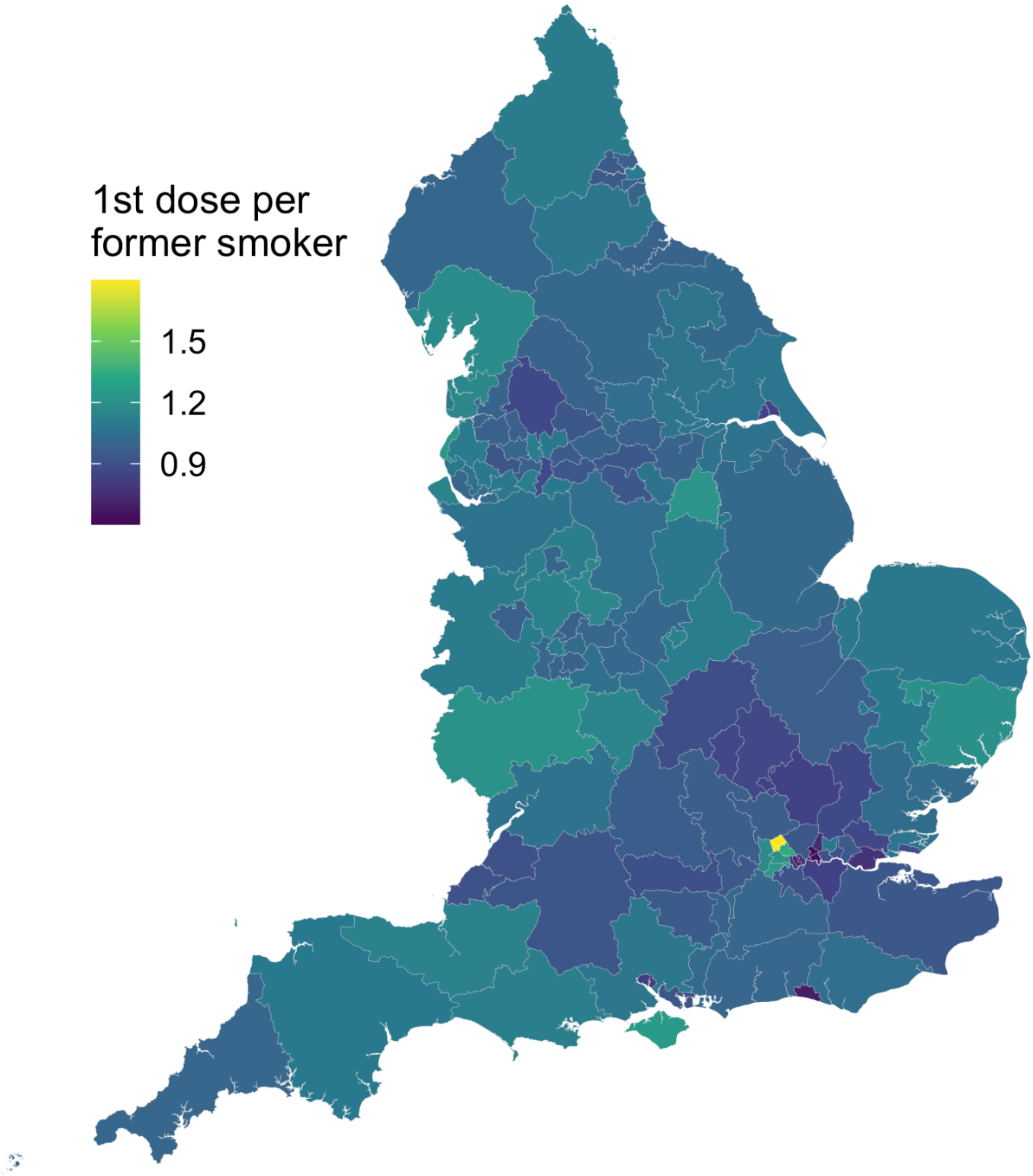

Supplement: S3 File — (PDF) [file pone.0259990.s003.pdf]
