# Supplementary figures and images for: Can vaccination roll-out be more equitable if population risk is taken into account?
Source: PLoS One. 2021 Nov 15;16(11):e0259990. doi: 10.1371/journal.pone.0259990 (PMC8592495; doi:10.1371/journal.pone.0259990)

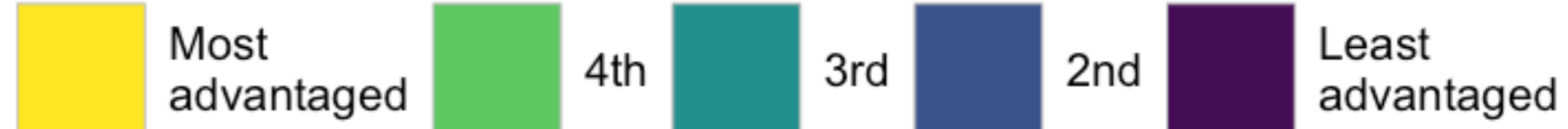

Area

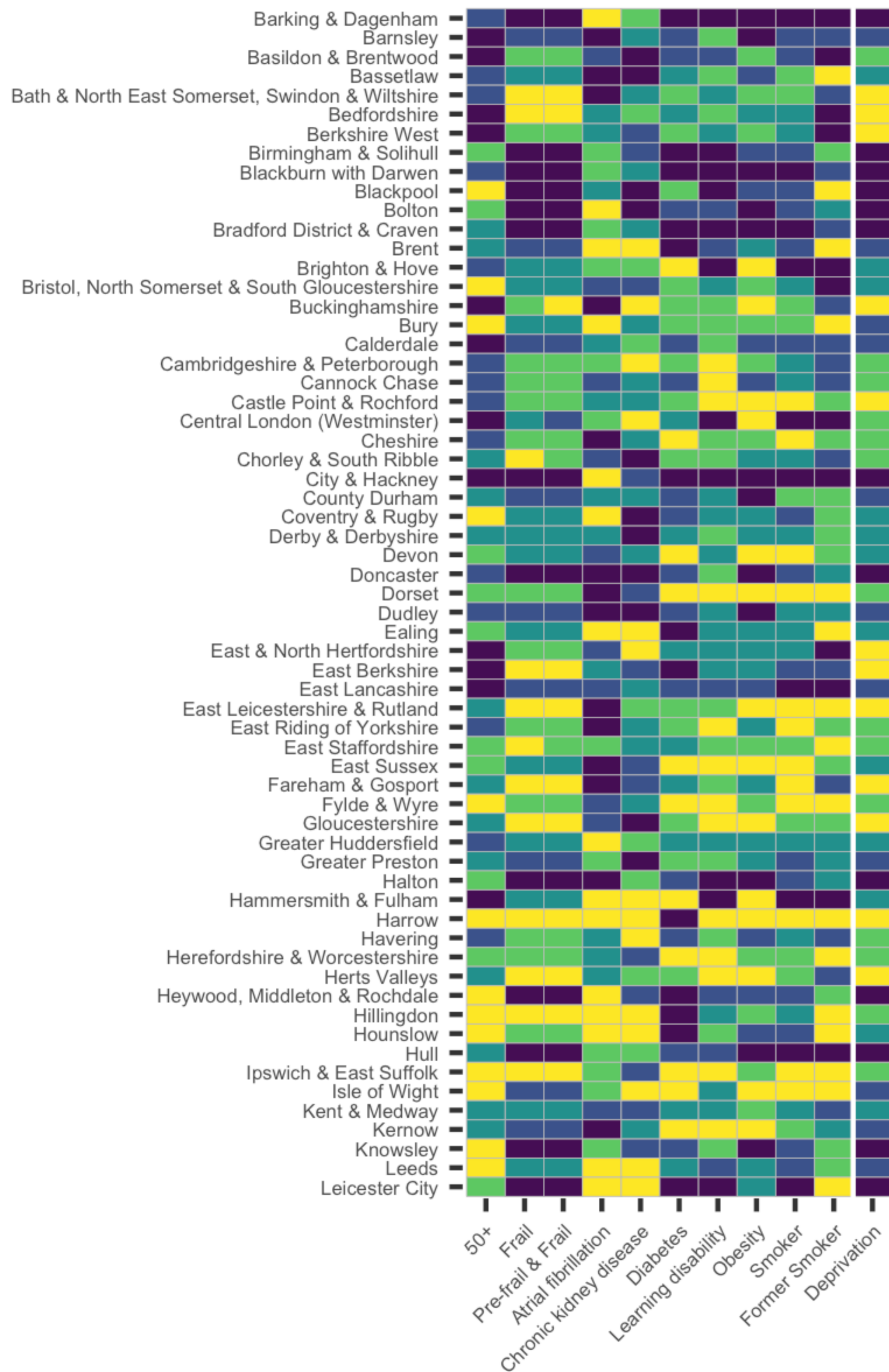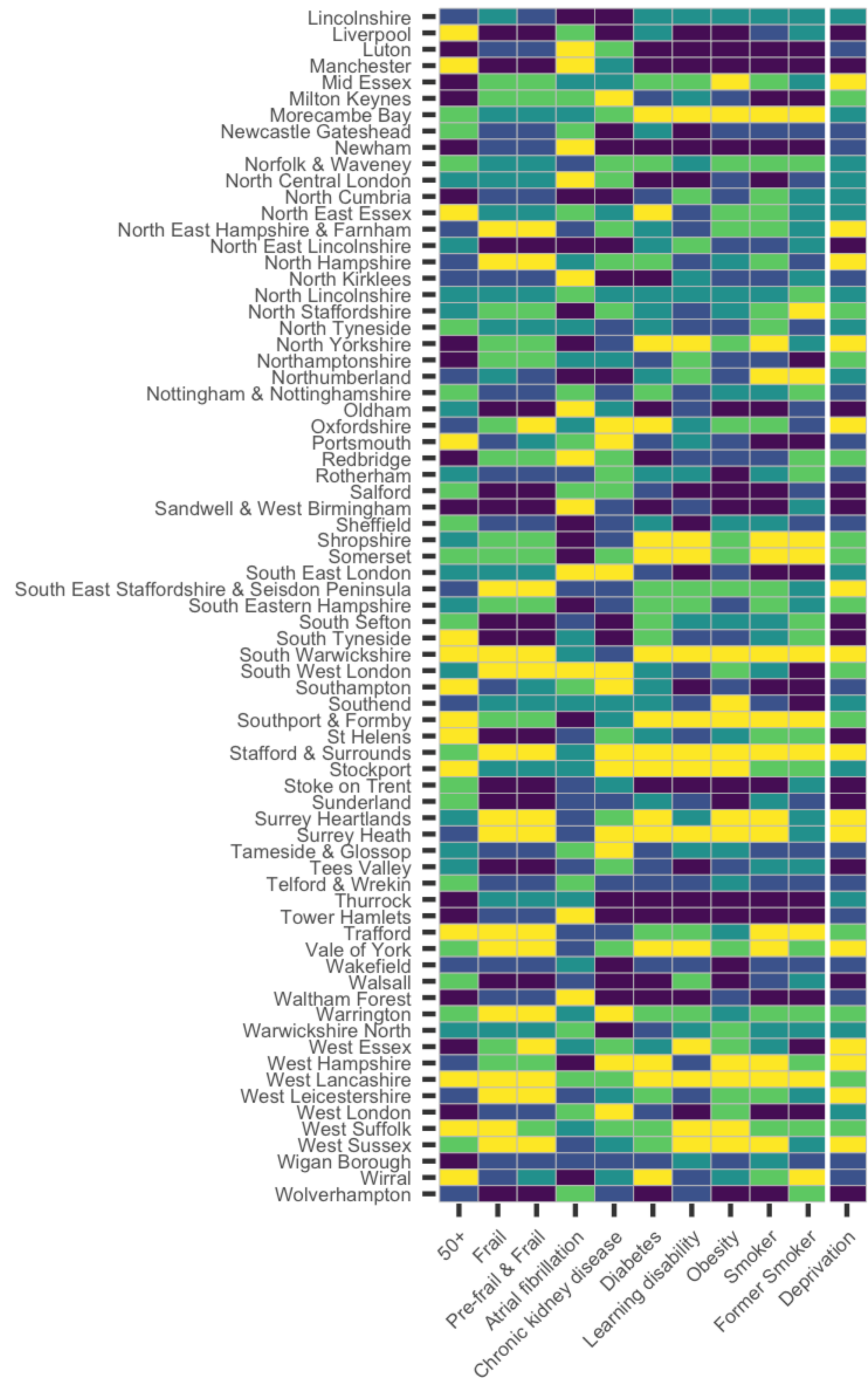

Supplement: S1 Fig — The risk factors are associated with infection, hospitalisation and mortality due to COVID-19. The ratio of first vaccine dose to the number of people with each risk factor (by quintile), in each area, are shown. Area deprivation quintiles (measured by the English Index of Multiple Deprivation) are also shown. All Clinical Commissioning Group areas in England are plotted. (PDF) [file pone.0259990.s004.pdf]
