# Supplementary material for: Can vaccination roll-out be more equitable if population risk is taken into account?
Source: PLoS One. 2021 Nov 15;16(11):e0259990. doi: 10.1371/journal.pone.0259990 (PMC8592495; doi:10.1371/journal.pone.0259990)
Supplement: Attachment — Submitted filename: Response to Reviewers.docx [file pone.0259990.s005.docx]

**Academic Editor comments**

1. *Please take into account Reviewer suggestions, to make the complex statistical background more easlily accessible to the readers, including the ones less familiar with UK NHS. In particular I would advice Authors to give more information regarding the calculation of Index of Multiple Deprivation and provide data regarding IMD quintiles in different CCGs, possibly adding a column in Figure 2, as suggested by Reviewer 2, and in Figure A4. (By the way, if I have understood well, the term “IMD quantile” used in Appendix should probably be replaced with “IMD quintile”). Moreover It should be better clarified how IMD was used at the individual level in ELSA and CFAS sample, to build the frailty prediction model.*

We thank you for these comments and suggestions. We have provided more detailed information regarding the Index of Multiple Deprivation calculation in the Population data subsection (lines 101-14 in the version of the manuscript without tracked changes). We have further included data regarding IMD quintiles in different CCGs in Figure 2 and Appendix Figure A3h. We have amended ‘quantile’ to ‘quintile’ in the Appendix. Finally, we have clarified how IMD was used at the individual level to build the frailty prediction model in the Frailty estimation subsection (lines 120-123).

2. *Moreover, as suggested by Reviewer 1, English policy regarding vaccination should better clarified (moreover ref 23 is no longer accessible on the internet). If priority groups changed after 25-FEB-2021 (and this seems to be the case according to*[*https://www.gov.uk/government/publications/covid-19-vaccination-care-home-and-healthcare-settings-posters/covid-19-vaccination-first-phase-priority-groups*](https://eur03.safelinks.protection.outlook.com/?url=https%3A%2F%2Fwww.gov.uk%2Fgovernment%2Fpublications%2Fcovid-19-vaccination-care-home-and-healthcare-settings-posters%2Fcovid-19-vaccination-first-phase-priority-groups&data=04%7C01%7CDavid.R.Sinclair%40newcastle.ac.uk%7C0351031e6c5645c5153108d96097b9dc%7C9c5012c9b61644c2a91766814fbe3e87%7C1%7C0%7C637647027060267783%7CUnknown%7CTWFpbGZsb3d8eyJWIjoiMC4wLjAwMDAiLCJQIjoiV2luMzIiLCJBTiI6Ik1haWwiLCJXVCI6Mn0%3D%7C1000&sdata=%2FKzpcN2R1L1egXk%2BXPUEk7L6xTSqMvQyGck2cozjc%2Fg%3D&reserved=0)*) this should be briefly discussed, as it seems to be consistent with Authors suggestions.*

Ref 23 (now 26) has been amended. The discussion of the vaccine priority groups has been expanded in the introduction (lines 63-66).

3. *As further suggestions, I feel that the aim (lines 87-89) should be more precisely stated and that the 10-year time interval between ELSA and CFAS data and the pandemic should be cited as a study limitation in the estimate of frailty.*

We have more precisely stated the aims in the Introduction (lines 91-93).

We agree that the 10-year time interval between ELSA wave 4 and CFAS II data collection and the pandemic is a study limitation. In view of this, we have re-run the analysis using data from ELSA wave 8 (2016/17) instead (and not ELSA wave 4 or CFAS II). This allows us to use the most up-to-date ELSA data for which deprivation-linkage is currently available. By dropping CFAS II we have also been able to expand the age-range in which we estimate frailty prevalence down from 65+ to 50+, allowing us to include younger frail population members.

This has slightly reduced the sample size, but the conclusions remain the same. The results using the more recent data are consistent with the previous results; frailty prevalence estimates for the CCG areas have slightly changed but the conclusions regarding vaccine inequities are the same.

**Journal requirements**

1. *Please ensure that your manuscript meets PLOS ONE's style requirements, including those for file naming. The PLOS ONE style templates can be found at* [*https://journals.plos.org/plosone/s/file?id=wjVg/PLOSOne_formatting_sample_main_body.pdf*](https://eur03.safelinks.protection.outlook.com/?url=https%3A%2F%2Fjournals.plos.org%2Fplosone%2Fs%2Ffile%3Fid%3DwjVg%2FPLOSOne_formatting_sample_main_body.pdf&data=04%7C01%7CDavid.R.Sinclair%40newcastle.ac.uk%7C0351031e6c5645c5153108d96097b9dc%7C9c5012c9b61644c2a91766814fbe3e87%7C1%7C0%7C637647027060317575%7CUnknown%7CTWFpbGZsb3d8eyJWIjoiMC4wLjAwMDAiLCJQIjoiV2luMzIiLCJBTiI6Ik1haWwiLCJXVCI6Mn0%3D%7C1000&sdata=DnfPNIZjzuSFpdkjuzEj9byrEIbSO9VvA4f9x0G%2F3Q4%3D&reserved=0) *and*[*https://journals.plos.org/plosone/s/file?id=ba62/PLOSOne_formatting_sample_title_authors_affiliations.pdf*](https://eur03.safelinks.protection.outlook.com/?url=https%3A%2F%2Fjournals.plos.org%2Fplosone%2Fs%2Ffile%3Fid%3Dba62%2FPLOSOne_formatting_sample_title_authors_affiliations.pdf&data=04%7C01%7CDavid.R.Sinclair%40newcastle.ac.uk%7C0351031e6c5645c5153108d96097b9dc%7C9c5012c9b61644c2a91766814fbe3e87%7C1%7C0%7C637647027060327528%7CUnknown%7CTWFpbGZsb3d8eyJWIjoiMC4wLjAwMDAiLCJQIjoiV2luMzIiLCJBTiI6Ik1haWwiLCJXVCI6Mn0%3D%7C1000&sdata=qBMfyxFI41oq6Me7O5FNteAR76rmyNRUOa5jeHtPfJY%3D&reserved=0)*.*

We have checked that the manuscript conforms to PLOS ONE’s style requirements.

2. *Please expand the acronym “NIHR” (as indicated in your financial disclosure) so that it states the name of your funders in full.
   This information should be included in your cover letter; we will change the online submission form on your behalf.*

We have expanded the acronym ‘NIHR’ in the financial disclosure. NIHR stands for the National Institute for Health Research.

3. *Acknowledgments Section: Move New Information to the Financial Disclosure:
   "Thank you for stating the following in the Acknowledgments Section of your manuscript:
   “DSi, FM, CW and AM who are funded by the National Institute for Health Research Policy Research Unit in Older People and Frailty (PR-PRU-1217-21502). DSi, CW and FM are also funded by the Integrated Covid Hub North East. DSt is funded by NIHR School for Primary Care Research (SPCR-PDF-2020-161). The views expressed are those of the author(s) and not necessarily those of the funders: Integrated COVID Hub North East (ICHNE), NIHR or the Department of Health and Social Care. CFAS II was funded by UK Medical Research Council (MRC; research grant G0601022) and the Alzheimer’s Society.”*

*We note that you have provided funding information that is not currently declared in your Funding Statement. However, funding information should not appear in the Acknowledgments section or other areas of your manuscript. We will only publish funding information present in the Funding Statement section of the online submission form.
Please remove any funding-related text from the manuscript and let us know how you would like to update your Funding Statement. Currently, your Funding Statement reads as follows:
“DSi, FM, CW and AM who are funded by the National Institute for Health Research Policy Research Unit in Older People and Frailty (PR-PRU-1217-21502;* [*https://www.nihr.ac.uk/*](https://eur03.safelinks.protection.outlook.com/?url=https%3A%2F%2Fwww.nihr.ac.uk%2F&data=04%7C01%7CDavid.R.Sinclair%40newcastle.ac.uk%7C0351031e6c5645c5153108d96097b9dc%7C9c5012c9b61644c2a91766814fbe3e87%7C1%7C0%7C637647027060327528%7CUnknown%7CTWFpbGZsb3d8eyJWIjoiMC4wLjAwMDAiLCJQIjoiV2luMzIiLCJBTiI6Ik1haWwiLCJXVCI6Mn0%3D%7C1000&sdata=S0X30Eupu6AB%2FQnEc2qqq4cU8uNIssBKjS%2FPN4KOMC0%3D&reserved=0)*). DSi, CW and FM are also funded by the Integrated Covid Hub North East. DSt is funded by NIHR School for Primary Care Research (SPCR-PDF-2020-161;* [*https://www.nihr.ac.uk/*](https://eur03.safelinks.protection.outlook.com/?url=https%3A%2F%2Fwww.nihr.ac.uk%2F&data=04%7C01%7CDavid.R.Sinclair%40newcastle.ac.uk%7C0351031e6c5645c5153108d96097b9dc%7C9c5012c9b61644c2a91766814fbe3e87%7C1%7C0%7C637647027060337477%7CUnknown%7CTWFpbGZsb3d8eyJWIjoiMC4wLjAwMDAiLCJQIjoiV2luMzIiLCJBTiI6Ik1haWwiLCJXVCI6Mn0%3D%7C1000&sdata=1zHCCIgn9PFGw5FvAqnYycSZW4lgjCs8OXG7NnmbTig%3D&reserved=0)*). The funders had no role in study design, data collection and analysis, decision to publish, or preparation of the manuscript.”*

Please amend the financial disclosure as follows:

DSi, AM CW and FM are funded by the National Institute for Health Research Policy Research Unit in Older People and Frailty (PR-PRU-1217-21502). DSi, CW and FM are also funded by the Integrated Covid Hub North East. DSt is funded by NIHR School for Primary Care Research (SPCR-PDF-2020-161). The views expressed are those of the author(s) and not necessarily those of *of the NIHR, the Department of Health and Social Care, or the* Integrated Covid Hub North

4. *We note that Figure 1 in your submission contain [map/satellite] images which may be copyrighted. All PLOS content is published under the Creative Commons Attribution License (CC BY 4.0).*

The digital boundaries and reference maps used in Fig 1 are sourced from the UK government. They are licensed under the Open Government Licence v.3.0 (<https://www.ons.gov.uk/methodology/geography/licences>). The Open Government License v3.0 is compatible with the Creative Commons Attributions License 4.0 (CC BY 4.0) (<http://www.nationalarchives.gov.uk/doc/open-government-licence/version/3/>)

**Comments from Reviewer #1**

1. *Please describe in more detail in the intro the current policy in the UK for vaccine distribution. In the conclusions the authors note that they use age, but this isn't clear. More detail is needed.*

Thank you for highlighting this. We have added more details on vaccine policy in England in the Introduction section (lines 63-66 in the version of the manuscript without tracked changes).

2. *Please add a heatmap of vaccines: patients with at least one risk factor to fig 1 as third panel.*

This has been added as Fig 1d.

3. *If you have data on COVID-19 infection rates (pre-vaccination) and/or mortality by county and could show if vaccine distribution/uptake maps to areas of higher cases and/or mortality that would also be of interest*

We agree that this is an interesting issue worth investigating, however the reasons behind vaccine hesitancy are beyond the scope of this paper and might be better considered in a separate study.

**Comments from Reviewer #2**

1. *I am very sympathetic to the argument that a more targeted approach based on frailty makes good sense. The age-based approach does have some practical advantages, in that age provides some approximation of risk and a much easier and non-arbitrary criteria to use in a large (and in many ways unprecedented) vaccination program rollout. The authors could expand on the practicality of using a frailty-based approach. Using the frailty index in England could be more feasible than in most other jurisdictions given the integration of the frailty measure in the Electronic Health Record which is (unfortunately) not a feature of most EMRs and health systems. Of course, it would be nice to aspire to bring other jurisdictions up to better including frailty measures in the health record rather than aiming for the lowest common denominator. Overall, it would be helpful for the authors to acknowledge both the pros and the cons of the age-based approach in more detail, with particular emphasis on practicalities.*

We thank you for this suggestion. As a results, we have expanded the Discussion to include these points (lines 227-242 in the version of the manuscript without tracked changes).

2. *In a related question, how would the use of a frailty-based approach work? Would it be done on an individual level where a person is invited to book an appointment based on their known frailty level or shows their frailty value in the vaccine clinic booking process in order to gain access to the vaccination? What about people who have not had access to health care to assess their level of frailty – would they run the risk of being passed over? Or would it be done at an area level where the population % with frailty would be considered and vaccines would be preferentially delivered to clinics in areas of high frailty to be then distributed in whatever way is most practical? This would also potentially have the benefit of creating a herd protection in areas of high vulnerability while keeping the rollout relatively less complex. In either case, it would be helpful to clarify which approach is being suggested.*

We have added to the discussion to consider this question (lines 244-251).

3. *The figures are helpful and interesting but I imagine they would be most easily interpreted by people with prior knowledge of social comparisons between these areas of England. Might the authors consider including socioeconomic status in the figures for easy reference? A third panel c could be included for the map showing the area variation in the Index of Multiple Deprivation for example in Figure 1. In Figure 2, another column could be added to each of the heat maps showing the IMD. The authors could consider whether this would help them enrich the context of their figures.*

We have added an area deprivation map to Figure 1 (Fig 1c). We have also added the deprivation quintile to Figure 2.

4. *This brings up the point that presumably the inequity in vaccine access arises at least in part because of socioeconomic status and including SES more prominently in the paper and figures would help make that clear. However, one wonders if this could be offset because in general lower socioeconomic status areas may have a higher density of frontline workers who may also have been prioritized in the early phases of vaccination. Indeed, it’s likely that a solid argument could be made that targeting vaccine uptake in lower socioeconomic areas would have a similar benefit to targeting areas with high frailty. The authors may consider strengthening their justification of why targeting by frailty is more important than targeting by SES in this way.*

This is an interesting point. We have included a paragraph to the Discussion that considers targeting vaccines by SES (lines 253-260).

5. *The use of the terms (in)equity and (in)equality could be clarified since they have different technical meanings.*

Thank you for highlighting this discrepancy. We have clarified this.
