# Supplementary material for: Can vaccination roll-out be more equitable if population risk is taken into account?
Source: PLoS One. 2021 Nov 15;16(11):e0259990. doi: 10.1371/journal.pone.0259990 (PMC8592495; doi:10.1371/journal.pone.0259990)
Supplement: Attachment — Submitted filename: Response to Reviewers.docx [file pone.0259990.s006.docx]

**Academic Editor comments**

1. *lines 159-161: add "as expected" to the following statement ("There is a clear negative association between the ratio of first doses to the number of frail people and the area deprivation").*

Thank you for this suggestion. We have added “as expected” to the above sentence, such that it now reads: “*There is a clear negative association between the ratio of first doses to the number of frail people and the area deprivation, as expected (Fig 1c)*" (lines 160-162).

2. *accordingly, modify discussion (lines 253-254), specifying that the present analysis model inherently implies a positive association between increased frailty prevalence and increased area deprivation, as area deprivation was used to estimate the number of frail subjects.*

We agree that it is helpful to make the relationship between area deprivation and frailty clear in the discussion. We are concerned that “*specifying that the present analysis model inherently implies a positive association between increased frailty prevalence and increased area deprivation*” may be misleading to some readers. The ordinal logistic regression fitted to the survey data does not necessarily imply a positive association between frailty and deprivation, rather this association is a result of the regression. This positive association is then imposed when estimating the number of frail subjects in each area.

Accordingly, we have amended this section (now lines 254-256) to read: *“Our analysis found a positive association between increased frailty prevalence and increased area deprivation. This positive association was imposed when using area demographics to estimate the number of frail subjects (Appendix 2).”*

3. *clarify statistical model (lines 124-125), stating that frailty category was used as outcome measure.*

Thank you for suggesting this improvement to the clarity of the manuscript. We have added that frailty category is the outcome measure of the statistical model in lines 124-125. The relevant sentence now reads: “*An ordinal logistic regression was calculated using each ELSA respondent’s age, sex and area deprivation quintile (IMD), with frailty category as the outcome measure*”.
